# State Provision of Resilience in Social Compulsory Care: A Vulnerability Analysis of Physical Constraint of Children and Youth Without Consent

**DOI:** 10.1007/s11196-023-09987-w

**Published:** 2023-03-18

**Authors:** Titti Mattsson, Sofia Enell

**Affiliations:** 1 Faculty of Law, Lund University, Lund, Sweden; 2 Department of Social Work, Linnaeus University, Växjö, Sweden

**Keywords:** Children’s rights, Resilience, Locked institutions, Sweden, Social services, Vulnerability theory

## Abstract

Children’s and young persons’ rights have received increasing been focus in recent decades, due in a significant degree to the UN Convention on the Rights of the Child. In Sweden, compulsory care in the social-services system is disputed, not least for the forceful measures that facility personnel have at their disposal to control children in certain conflict situations. The general aim of this article is to examine how the increased emphasis in Sweden on children’s rights is promoting resilience for children and youth in youth compulsory secure-care settings. A more general question is whether the child-rights discourse leads in practice to increased resilience for children and youth in this setting, or even in general. The empirical material shows that children and young people’s perceptions of care and treatment are strongly linked to their interactions with staff and how the staff use restrictive measures. Applying Martha Fineman’s vulnerability theory in this context means that achieving resilience demands an analysis of the institutional settings in which children and young persons live their day-to-day lives, including their relationships in this setting. Comparing the legal possibilities of physical constraint with interviews of children and personnel reveals that relevant legislative frameworks and children’s-rights discourse should serve as a protection mechanism for children and youths, but in real life, these seem to have limited effect.

## Introduction

George Floyd’s killing in the United States in 2020 highlights in an extreme way the rare but possible risks of harmful behavior toward individuals by law enforcement or other persons acting officially on behalf of the state.^1^ The potential problem of state officials harming individuals, intentionally or unintentionally, is also a recurring theme in countries such as Sweden. For example, since 2000, increased focus has been directed at personnel behavior in the social care of children and youth^2^ in Sweden [1, 2]. While nothing as extreme as the George Floyd tragedy has occurred in this country, several incidents (ranging from derogatory attitudes or ignoring to physical violence and sexual advances) have highlighted the ever-present risk of harmful behavior towards young persons in secure-care settings (locked institutions). The situations also highlight the need to find legally and empirically supported methods and ways of achieving resilience that personnel can use in stressful situations and when necessary (for example due to the youth’s own health or safety). At the same time, personnel actions that are not clearly part of a legally enforced decision according to legislation may infringe on children’s and young people’s fundamental rights.

The increasing focus on children’s rights in recent decades is largely due to the UN Convention on the Rights of the Child (hereinafter referred to as the CRC) [3–6]. Like many other countries, Sweden has implemented the convention to domestic legislation [7]. The child rights discourse has taken place and continues to do so both on national policy levels and in different public activities, including social services, that directly or indirectly affect the everyday lives of children and young persons. Incorporation of CRC into domestic legislation, as requested by the government, is intended to ensure that children’s rights become a central matter of concern for everyone. In this way, politicians as well as professionals in areas such as healthcare, law enforcement, schools, and social services are expected to apply the CRC in their daily work. As a result, since the CRC’s incorporation into law, Sweden has witnessed recurring discussion regarding what needs to be done, in legislation and in practice, to satisfy and strengthen the agendas, safety needs, and best interests for children and young persons. One primary area of concern has been *secure-care settings for children and young persons* in Sweden, including the matter of how to ensure that all officials act in a non-harmful manner towards the children and youth who are placed in these institutions [2, 8].

The general aim of this article is to examine the extent to which increased emphasis on children’s rights in Sweden is promoting resilience for children and youth in secure-care settings. Using the terminology from Martha Fineman’s vulnerability theory—where vulnerability is viewed as the primal human condition for everyone—the term *resilience* stands for what may be done during our lifetime through various social institutions and relationships to diminish a person’s vulnerability [9, 10]. One central question, then, is this: *To what extent*—*and how*—*is resilience for children and young persons in locked settings promoted and obtained through the non-harmful behavior of staff*? The more overarching question is whether the child rights discourse leads in practice to increased resilience for children and youth in this setting, or even in general. One way to start examining these questions is to analyze a legal gray zone, defined in this context as “a situation in which it is not clear whether something is legal or illegal, acceptable or not acceptable” [11]. In this article, the use of so-called physical constraint (holding, in Swedish: *fasthållning*) will be used to analyze the connection between children’s rights and resilience for children and youth in Swedish secure-care institutions. Physical constraint of children and young persons in a secure-care setting is a legal gray zone in Swedish compulsory care, and therefore provides a suitable example for such analysis. For this case, relevant legal frameworks, key government decisions, and voices of young persons in secure-care institutions will be studied.

The analysis in this chapter applies Fineman’s vulnerability theory [9]. Young persons in secure-care institutions are often trapped in their categorization as a “vulnerable group,” as this concept is traditionally understood. The Western legal tradition assumes a subject that is independent, self-sufficient, and able to pursue his or her own interests and achieve his or her goals. Transferring this ideal to children and youth in institutional care creates a risk that they may be viewed as a stigmatized group and thus vulnerable, irrespective of their individual abilities [12, 13]. The risk is that they are described with certain stereotypes of what children and young persons in institutional care are like, and what they need. To conceptualize such children and young persons in institutional care as a group, therefore, ought to be problematized. Instead, the State needs to respond to the needs of all its people. This highlights the State’s obligation to equip persons with the resilience necessary to deal with their inevitable vulnerabilities [10]. In setting examined in this article, these prerequisites can include good relations with the facility staff, a safe environment, and chances to heal and grow as a person. Responsibility for ensuring that these conditions are met includes regular review of legislation and practices, along with listening to the voices of persons affected by various forms of state intervention (here, children and young persons). Further, according to vulnerability theory, the only acceptable state actions are those designed to ensure that state power is used to enhance the resilience of others—in this case, children and young persons. In other words, the goal is an active State that emphasizes citizens’ individuality, interdependency within social institutions, and the need for public authority to provide those citizens with resilience.

The theory also assumes that *equal* opportunity can be achieved only by offering *different* ways in which situations of dependency can be overcome by legislation or practice [14]. In line with this assumption, the theory also emphasizes the need for respecting difference and diversity among persons and during persons’ lifespans (28). Even though the theory emphasizes the complexity of human needs, it also highlights universal concerns for every human being, such as influence and dignity.

With this theoretical approach, one central issue concerning social child welfare care is the extent to which—and how—resilience can be promoted and obtained for children and young persons in locked settings. A more general issue, which will be touched upon in this chapter, is the extent to which increased emphasis on children’s rights in legislation is a suitable means for promoting such resilience. Finally, by choosing a legal gray zone to study, we wish to highlight the need of the State to work proactively to ensure that the borders between accepted and non-accepted actions towards children and young persons in locked institutions are clearly defined and in line with children’s rights according to the CRC. This to ensure that children and young persons in locked institutional care will not be subject to harmful behavior outside the realm of necessary interventions for care purposes.

The paper is structured as follows. After this introduction (1), we provide a brief background description of Swedish social welfare services and state-run secure-care institutions, and the legal conditions for placement in such institutions. (2). Thereafter the case of physical constraint is presented, including relevant legal frameworks, government practices, and the voices of young persons in secure-care institutions. (3). Finally, we analyze our results using the vulnerability theory and make some final conclusions (4).

## Locked Institutional Care for Children and Youth—a Background

In an international perspective, Sweden’s locked institutional care stands out. A comparison of ten different (Western) countries revealed that Sweden has the highest proportion of children placed in temporary *seclusion* [15].^3^ The proportion of children in this type of care in Sweden (31.1 per 100,000 children) is about three times higher than in Denmark, England, Scotland, Wales, and Northern Ireland.^4^ In addition, the majority (almost 90 %) of children placed in temporary seclusion in Swedish secure-care institutions have been placed there as accordance with the national child-protection system. This practice differs from countries such as England, New Zealand, and Norway, where children and youth are detained mainly through the criminal justice system. Even in a Nordic context, Sweden can be viewed as unique. Unlike social child-protection systems in the other Nordic countries, seclusion and restrictions of rights and freedoms within Swedish social child protection are carried out only in secure care institutions [17].

The main rule is that the care of children and youths should be provided primarily in voluntary forms under the Swedish Social Services Act (2001:453). According to the Social Services Act, the public social-service system shall encompass all people in Sweden who are in socially or economically in need of help or assistance. It is a municipal responsibility, and the regulation embraces all persons who reside in or are visiting the municipality. The overarching goals of the Social Services Act are to promote economic and social security, equality in living conditions, and active participation in the life of the community. Further, the social services shall work to liberate and develop the innate resources of individuals and groups. When measures affect children, the best interests of the child should be given special consideration. The Social Services Act stipulates that all activities are voluntary for the recipient and should be based on respect for that person's self-determination and privacy.

In certain situations, however, compulsory care may become an option for the social services. The conditions for compulsory care of children and young persons are set out in the Swedish Care of Young Persons Act (1990:52). One condition for triggering this care is lack of consent from the child’s legal guardians or, if the subject is older than 15 years, the young person him- or herself. The other condition is the existence of a significant risk for the child’s or the young person’s health and development. This risk can be related to circumstances in the child’s home or to the young person’s own behavior. Compulsory care in Sweden requires a judgment issued by the National Administrative Court.^5^

Children and youths who are considered to need particularly close supervision are often placed in facilities by the National Board of Institutional Care (SiS) [18].^6^ The National Administrative Court is not involved in this case; social services makes SiS assessments and decisions. At these (mainly locked) institutions, there is a legal possibility to restrict children’s and youth’s lives by exercising several restrictive measures stipulated in the Care of Young Persons Act. Examples such measures include authority to determine use of solitary confinement, care in solitude, bodily search and external physical examination, prohibition of contact with people outside the facility, and limitations on participation in certain activities within the institution.^7^ Decisions regarding restrictive measures are made by institution staff, but the subject’s legal guardians or a subject older than 15 years can appeal most of these decisions can be appealed to the National Administrative Court. There is no lower age limit for placement in a secure-care institution, but the maximum subject age is 21 years; most young persons placed in such care are 15 to 16 years old, but children as young as 8 years have been placed secure-care institutions. While a majority of these young persons are boys, one third of the subject are girls [19]. The young persons placed in secure-care institutions often have a complex combination of problems such as delinquency and substance abuse, together with difficulties at school, family conflicts, experiences of abuse, and friends with high-risk behavior.

Thus, this form of care allows specific restrictions on freedom of movement; the accompanying risk is that actions will be taken that violate the young person’s right to personal and physical integrity. Young persons in secure institutional care may thus find their rights severely curtailed under the law. Even if the restrictions are based on subjects’ needs for care and treatment and a belief that they pose a danger to themselves (and sometimes others), loss of the most basic rights can render their status as rights holders even more dependent on the fulfilment of resilience by others. This also means that young persons in locked institutions are essentially at the mercy of a group of staff members working under a single management—what Goffman called a “total institution” [20]—with far-reaching powers at their disposal.

## The Case of Physical Constraint

### Introduction and Legal Framework

A central question that is raised when discussing the risk of harm in a secure state care setting is whether there are measures taken by institutions and their employees that are partially or completely unsupported by law [see also 21. The importance of legal support for physical interventions against young persons in compulsory care is fundamental from a legal security point of view and a child rights perspective. This requirement on clear legal support for any personal intrusion during public care also highlights the ever-present need to strive for methods and ways to achieve resilience during contact with the state.

However, analysis of applicable legal material indicates that there may be a discrepancy between how Swedish legislation stipulates the use of physical interventions with children and youths in compulsory care at secure-care institutions and how these restrictive measures are used in practice. This breach between theory and practice particularly concerns the phenomenon of physical constraint.

*Physical constraint* is usually defined as restraint with physical force, e.g., a scenario in which personnel hold a young person’s arms and legs to restrain them. Physical constraint is not regulated in the Swedish Care of Young Persons Act (1990:52) and therefore is not a formal ground for a legal decision concerning social child-welfare care according to regulation. However, in practice, it is still used, for example when a young person attempts to leave a secure-care institution, when a fight or quarrel occurs, or when a youth is taken to solitary confinement (temporary placement in an empty room under the observation of staff, in Swedish: *avskiljning*). In practice, physical constraint is often used to enforce a solitary confinement. The closest legal support for restraining a child or youth in this manner is found in Chapter 24, Sect. 2 of the Swedish Penal Code, which regulates the legal authority to use force in certain situations in general [22].^8^

### Government Reports and Parliamentary Ombudsman Cases

A governmental report from 2015 [20] discusses whether there is reason for regulating physical constraint in the Swedish Care of Young Persons Act. One argument in favor of doing so, according to the report, is that *physical constraint* (fasthållning) can be regarded as being equally intrusive as, for example, *solitary confinement* (avskiljning). It is argued that on the one hand, regulation would allow improved scrutiny of physical contraint’s use from a procedural-guarantee perspective. However, the report concludes, problems may arise in relation to the supervisory responsibility of staff in other forms of residential care than secure-care institutions. Therefore, according to the report, physical constraint should continue to be seen as part of the *enforcement* of existing restrictions and not an action on its own [20].

The Government agrees with the inquiry’s assessment, but stresses that SiS must work to develop methods for avoiding coercive measures to the greatest degree possible and for dealing with situations in ways other than physical constraint. An example of such methods is SiS’s conflict management program, *No Power No Lose* [23]. The program teaches techniques for keeping youths in a non-reactive state, in a specific location, without force and as safely as possible. The program includes training for personnel on how to recognize early signs of violence and use of verbal de-escalation in conflicts, as well as physical techniques to move young persons safely and securely in the event of violent behavior at the institution. However, because physical constraint is an intrusive form of physical intervention, it cannot be appealed by the youth involved, and because it is associated with risks of injury, the measure should be used very restrictively [24].^9^

The No Power No Lose conflict program played a central role in a study of how solitary confinement was used at the secure-care institutions run by SiS [25]. Swedish nonprofit children’s rights organization Barnrättsbyrån conducted the investigation and found that in at least 80 percent of all decisions to use solitary confinement, personnel used various physical techniques to transport, hold, or place children and youths in a prone position as part of carrying out the measure.^10^ Despite the authority’s training and emphasis on using verbal de-scalation techniques, staff often used violence first, prompting the young person to become violent, and resulting in physical constraint for the young person. In a third of all decisions on solitary confinement, the young person was only physically constrained and was never taken to the room designated for solitary confinement. In some cases, there did not even seem to be an intention to get the young person to the designated room. Overall, the study addresses the severe lack of non-violent management of situations where young persons are angry or upset, but not necessarily violent.

Situations in which physical constraint was used on youth at secure-care institutions have also been the subject of Parliamentary Ombudsman's inquiries in Sweden. In a decision that has received particular attention, the Ombudsman criticized personnel at a youth care institution for violating a young person’s constitutional freedom from forced bodily interference through acts taken towards a youth [26].^11^ According to the Ombudsman, medical records indicate that the staff forced the youth into a prone position and held him/her on the floor *as an act in itself*, not as a step in fulfilling an earlier decision to apply solitary confinement. The Ombudsman points out that there is no legal support for such action in the Swedish Care of Young Persons Act without a formal decision to use solitary confinement. The absence of a solitary-confinement decision therefore reduces the possibility for transparency and legal security for the youth, because there is no decision that the youth can appeal [24].^12^

### Complaints by Children and Young Persons to the Health and Social Care Inspectorate

What, then, do care-facility personnel and youths think about physical constraint decisions in practice at the secure-care institutions? One way we have approached this question is to examine 50 cases selected randomly from approximately 300 complaints to the regulatory authority Health and Social Care Inspectorate (IVO) during 2012–2021. IVO is a national authority that handles complaints regarding serious flaws or deficiencies in Sweden’s social-services system.^13^ Some of these cases involve physical constraints at secure-care institutions. From the young persons’ perspective, a conclusion seems to be that the actions taken by the personnel cause uncertainty and pain. On the other hand, personnel in some of these cases, found it difficult to determine when a physical constraint should be considered a decision concerning solitary confinement. The personnel’s perspective is mirrored by Barnrättsbyrån’s results showing how often physical constraint was used without subsequent moving the young person to solitary confinement [25].

In one case, a youth complained that the staff at the institution sometimes grasp subjects very hard when a young person does not do as told and that staff often use the wrong grip when they restrain youths.^14^ In another complaint from a youth at a secure care institution, the youth stated that when being restrained by personnel, he/she had been treated harshly, could not breathe, and thought he/she was going to die. The youth also stated that he/she still felt bad about the situation.^15^ In a third complaint, a youth reported that the personnel had wrestled and locked his/her arm, causing pain.^16^ In this case, IVO criticized the institution for discrepancies in information about the reasons for the physical constraint: whereas personnel on duty describe the physical constraint as solitary confinement of the young persons, the officer on call claims the physical constraint was merely to prevent further violence from the youth.

### The Voices of Children and Young Persons

In a study of the practices of children’s and young persons’ rights in secure-care settings, we interviewed 15 young persons (five girls and 10 boys) aged 13–19 years.^17^ At the time for the interviews, these youths were placed at four different secure-care institutions. All of them were in compulsory care and most of them had extensive previous experiences of placement in foster care as well as residential (open) and secure (locked) care. For some, the placement at the secure-care institution was their first experience of out-of-home care.In individual and semi-structured interviews, the young persons were asked about everyday life at the unit, how they understood treatment in secure care, and their rights during the placement.^18^ All interviews were recorded and analyzed using thematic analysis [27].

One overarching theme in the interviews was the young persons’ relations to the staff. On the subject of staff-care-recipient relations, the young persons also talked about restrictive measures, violence on the part of staff, and their experiences of reporting abuse and appealing decisions on restrictive measures. Below, we present children’s and young persons’ voices about staff relations, experiences of staff violence, and reporting of abuse and appeals on decisions.

#### Young Persons’ Relations to Staff

When children and young persons talked about their everyday life, their treatment, and their rights in a secure care setting, they emphasized the ways in which staff treated and cared for them. They described how staff listened to them and treated them with respect, but also how staff fell short. In cases where subjects experienced staff as supportive, youths talked about staff being there for them in their everyday life at the institution, especially when youth were not feeling well. David, a 15-year-old boy, described how his keyperson (a staff member assigned to maintain regular contact with the young person during placement at the institution) gave him guidance and encouragement, and how they had informal conversations about things that were important to David.

We talk about things that have happened, and things that are going to happen. And he tries to help me, give me energy in a way that makes me continue to do what I’m doing. […] we go to the break room, we have a cup of coffee, and talk. We sit down and talk for like 3 min, 5 min. It’s also a talk. We talk about important stuff, everything, we talk about something important. (David, age 15)

While some young persons shared David’s experiences, most of them also mentioned staff members who failed to listen and who did not show respect for them and their situation. Although these young people shared experiences of staff members that appeared engaged in their work as well as those who did not care about them, it is often the stories of not being heard or being devalued by staff members that young persons gave the most space and importance. Melissa, a 16-year-old girl, shared her experiences of placements at several secure care institutions. She claimed that some staff use their power simply because they can:

And I think that’s really hard because every little thing that happens, it’s always like—“if you do this, I can do this and then you cannot do anything”. And you feel small, very small. And you don’t know what to do. I’m used to it. (Melissa, age 16)

Melissa said that owing to her experience of about 10 placements at secure-care institutions, she had become accustomed to being told that she had no say and that the staff would make the final decisions. Feelings of powerlessness are shared by the youths and were related to the fact that staff had the authority to use physical force. Hamza, a 14-year-old boy, shared his experiences of staff at a secure-care institution from a previous placement. According to Hamza, the staff cared only about those whom they liked, and they became violent if their orders were questioned—something Hamza could not refrain from doing.

But still, you have to stand up for yourself. Do you understand? Then they push the alarm and then fuck, they take you to isolation [*the room for solitary confinement*]. There is nothing else. But here [*present secure-care institution*], there are no abusers of power. (Hamza, age 14)

The youths’ stories about how they were treated by staff and the importance they placed on this aspect can be understood in terms of the very skewed distribution of power between the young persons and the staff. Since staff have the authority to take physical measures to restrict youths’ rights and freedom, the youths are dependent on the staff when it comes to having a tolerable life situation while placed in the institution.

#### Staff Violence

A few young persons share more detailed stories about staff violence in secure-care institutions. When Melissa were asked to explain why she was assigned her current placement in a secure-care institution, she said that she was violent and offensive. However, she noted that she became this way during her previous placement at another secure-care institution.

Melissa: I used my anger outwards because I learned that that’s what I should do when I was there.

Interviewer: At [name of secure institution 1]?

Melissa: Right, there were a lot of violence between the staff and the youths, and there were fights all the time. Not a very calm environment, it was not a safe place to be. (Melissa, age 16)

Melissa’s experience of the unsafe and violent institution (which was subsequently closed because of severe abuses by staff) was in the past, but for Jonas, the violence was in progress, at the institution in he was placed at time for the interview.

Well, I’ve seen many who have been beaten by staff. They are allowed to take us down, you know. But a lot of them have boxed and such, I mean they’ve beaten the youths. (Jonas, age 17)

Jonas explained that staff were “playing with us” and could continue to do so; all staff members wear alarms, and view themselves as worthier and on a higher level than the youths. When Jonas were asked how he coped with the staff’s illegitimate use of violence, he replied: “I don’t care that much. I come from a tough environment, so I don’t care about that”.

Melissa and Jonas seem to relate to their experiences of violence at the institutions in different ways. While Melissa learned to use violence, Jonas thought his hardships during his childhood made him more tolerant towards the violent behavior in institutional settings.

#### Reporting Abuse and Appealing Decisions

Some of the youths dealt with violent or unjust actions by exercising their legal right to complain or appeal restrictive-measure decisions. One of these was Lea, a 17-year-old girl. She explained how she reported incidents to the Parliamentary Ombudsman.

Yes, I made a report… that I … well I can tell you the whole situation. It was an evening, and we were about to go to bed, and I quarreled with one of the staff members. And I said like … I said ugly words in Russian to him because he was Russian. And then he became very angry. Then he took me and threw me on the bed and laid down on top of me. I reported that situation, but it never went through. Well, and then I moved from there and then …well, the report never went through, I haven’t heard anything since then. (Lea, age 17)

At time for the incident that Lea reported, she was younger than 17, but in her story, she did not talk about being transported to solitary confinement—a decision that could have legitimized the staff member’s physical actions. Nor did she say anything about staff helping her to make the report or letting her know how her case was progressing. Not getting any information about a complaint was something that Clara, another youth, also experienced. In her interview, as in many of the other interviews, she described how the staff informed her about her right to appeal a restrictive-measure decision. Clara did not experience this as being sincere. Based on this impression and previous experiences of appealing restrictive-measure decisions, she no longer makes any complaints or appeals.

Just, “This is the decision; you can appeal it if you want to”. And I just said, “Okay, what happens then?”, and they said, “Well nothing really, there is no one that will resolve this, nobody cares”. And I said, “So it’s just so you can feel that you have a say?”, like. […] I used to appeal everything, but I never got anything back, I haven’t gotten anywhere. (Clara, age 19)

Clara was not alone in concluding that appeals and complaints are meaningless. Melissa and Jonas also said that staff always informed them about their right to appeal, but because nothing ever comes of it, they have both given up. Jonas claims: “I think it’s just a waste of time”.

Thus, the interviewed young persons at the four secure-care institutions in this study emphasized the importance of how staff treat them, especially when decisions on restrictive measures are made. They perceived that staff use physical violence to demonstrate that they have the power. Their experiences of making complaints about staff misconduct or appealing a decision they find unjust is that no one listens to them. Their experience of being rejected stems from inaction: they are not informed about the progress or outcome of their complaint or decisions are not changed in their favor.

### Concluding Remarks

It is difficult to draw conclusions about specific measures taken at secure-care institutions, as each measure is based on a case-by-case assessment. However, it can be generally concluded that the difference between the attitude of legal actors towards physical constraint and the extent to which it occurs in “reality” forms a gray zone for the situation of youths placed in these institutions. The statements made by legal actors against physical constraint are thought to serve as a protection mechanism (albeit small) for the youths, but in real life these statements seem to have very limited impact.

In the light of what the legislator, SiS and the Parliamentary Ombudsman have stated about physical constraint, it seems that constraint is something that should not be used in a secure-care setting in its current form and status. SiS has stated that physical constraint is the most intrusive form of physical intervention, and the Parliamentary Ombudsman has severely criticized ecure-care institutions whose personnel have used physical constraint outside the context of solitary confinement. In addition, according to the regulatory framework (or lack thereof), physical constraint should not be used in state-run secure-care institutions. Nor should it be an alternative to solitary confinement. At most, it is possible that physical contraint should part of the enforcement of a solitary-confinement measure.

Listening to the voices of young persons in care illustrates just how central the relationships and interactions with facility staff are for these young persons. The way they are treated by staff seems to determine how young people experience their stay in a secure-care institution. From this perspective, the staff’s far-reaching powers are ever-present; the youths’ care experiences are a function of how staff relate to and use the restrictive measures they have at their disposal. In their accounts, young persons share experiences of being abused or seeing others being abused by staff, as well as experiences of being let down by the legal system through insufficient information about the process itself and the outcome of their complaints.

In sum, despite clear messages from the Swedish authorities that physical constraint should be used very sparingly and *only* in the context of solitary confinement, it still seems to be accepted by staff as a restrictive action in secure-care institutions. Consequently, the risk of being exposed to (direct or indirect) violence seems to be constantly present to youths in secure-care institutions and these young people have an uncertain legal status related to being subjected to violent actions by staff.

## Analysis and Discussion

The overall question of this article is whether—and if so how much—the current child-rights discourse in various policy-making areas concerning children is leading to increased resilience for children and youth, and whether other measures are needed to achieve this resilience. Using the example of Sweden and its restrictive measures towards children and young persons in the form of “physical constraint”, we wish to examine the extent to which developments are currently promoting resilience for children and youth in secure-care settings for youths.

For this case study, we analyzed relevant legal frameworks, key government decisions, and voices of young persons at secure-care institutions. From the analysis of the legal material, it has become clear that physical constraint is currently a “gray zone” in the situation of youths placed in these institutions. The law is unclear, and practice seems to vary widely from institution to institution.

With Fineman’s vulnerability theory as our theoretical approach, we examined one central issue: *to what extent and how can resilience for the children and young persons in locked settings be promoted and obtained*? The first issue, then, is the means through which such resilience can be reached.

The traditional answer to such an inquiry is often found by referring to criteria for achieving legal security in a traditional sense. Consequently, a traditional answer to the question of how to achieve resilience would most likely focus on criteria such as clear and admissible legislation and practice (including appeals when the subject is not content with the decision), where both the law and execution of the law offer equal opportunities without discrepancies based on gender, class, age, or other discriminatory factors. These are of course desirable goals. However, in reality, such an approach is seldom enough to achieve resilience for all individuals; in this case, children and young persons are often not considered full legal subjects and therefore they are not seen as having full legal capacity status to act in all legal settings. Further, the range of action is often very limited for a young person in institutional care, where many decisions are not formal or possible to bring before a court. Thus, a complementary approach to achieving resilience for these young persons is desirable.

As mentioned above, the theoretical framework for this chapter is the so-called vulnerability theory developed by Martha Albertson Fineman. Vulnerability theory provides an alternative approach to a rights-based paradigm for understanding concepts of state responsibility and dependency. It illustrates the constant dependence on human relationships and societal institutions that operate justly and that balance the interests of all. Vulnerability theory is regarded internationally as one of the leading critical approaches within welfare law. This theory provides critique on the prevalent social-liberal discourse, with its focus on individualism, autonomy, and independence. The idea of the theory is instead that individuals, states, and societal institutions are fundamentally vulnerable entities [28]. Therefore, we should not structure society based on independence—whether for the state or the individual person—as the norm. Instead, we ought to take a holistic view, and create sustainable institutions and arrangements for cooperation that can better deal with the many vulnerabilities common to a welfare state and its citizens, young or old. Doing so will enable us to achieve both durability and resilience—in the sense of an ability to recover and thrive—and thus create good prospects for achieving fair and decent living conditions for the population of a state, including its youth. According to Fineman’s vulnerability theory, only actions guaranteeing that state power is used to enhance the resilience of others—here, children and young persons—are accepted. Thus, the uncertain legal situation and questionable practices of physical constraint discussed above seem highly problematic from a vulnerability perspective. The unclear borders between accepted and non-accepted staff actions towards children and young persons in secure-care institutions are not in line with the goal of achieving resilience.

The uncertainty surrounding the limits to what staff can do towards the youth in Swedish secure-care institutions does more than put the goal of resilience at risk. It also signals the failure to fulfil children’s rights according to the CRC. For example, according to Article 3, when interacting with a child in a state-run secure-care institution, personnel should primarily consider the best interests of the child. According to Articles 19 and 20, the state has the responsibility to act with legislative and other measures to protect the child from all forms of physical or mental violence when in a care setting and, if needed, the state must offer special protection and assistance.

A general issue that must be discussed in this context is the extent to which increased emphasis on children’s rights in legislation is promoting resilience in practice. In this past 10–15 years, Swedish authorities working with children and youth have had a responsibility to realize children’s rights in various sectors of the national community. For example, in 2014, SiS decided upon a new action plan for the authority—based on a national strategy to strengthen children’s rights in Sweden that the Swedish Parliament adopted in 2014/15. Since 2017, SiS has worked continuously together with the national Ombudsman for Children and with other national authorities in Sweden to improve compliance with the Child Rights Convention.^19^ During our interviews with personnel at secure-care institutions, it also became clear that all institutions are working continuously with both employees and children to deal effectively with child-rights issues; results include guiding institutional documents for how to implement a child-rights perspective. Similar trends can be seen in every corner of the national stage, in municipalities, regions, schools, and other areas.

Though many improvements to increase knowledge among personnel and children have been achieved through programs and education on the topic, this question remains to be answered: to what extent can the child-rights discourse substitute other discourses? Can this approach replace other ways of thinking about the relationship between children, adults, and society? In this context of institutional care, it seems clear that secure-care institutions must avoid critical thresholds such as harmful behavior and strengthen their capacity to cope with the stresses caused by the challenging environment of secure care. Taking children’s rights into consideration seems to be of primary concern. At worst, the issues at stake for the child include the risk of physical abuse, deprivation of liberty, and psychological harm.

The risk of relying solely on a rights discourse is missing a central goal for the child, institution personnel, the institution, and society: resilience. Resilience can take many shapes and the right to go to court to challenge a government decision can sometimes be a powerful tool for achieving this goal. However, for children and young persons, a major problem is the limited possibility to exercise their rights in practice and the limited tools that young people may apply in court. For example, the result from a case study in our project concerning appealed decisions at SiS secure-care institutions shows that the possibility for children and young persons to succeed in an appeal against a solitary-confinement decision is almost non-existent [29].^20^ The young person appears to be at an evidential disadvantage in relation to SiS, because the courts base their decisions almost exclusively on information provided by SiS—even if the burden of proof is on SiS and the young person offers a different account of the events. Because young persons’ chances of success in court appear to be non-existent, one may argue that a substantive right of appeal is lacking. From a vulnerability approach, one can argue that there is a flaw in the design of this system. Some of the consequences of these flaws of the system can be identified when young persons share their experiences of making complaints or appeals in secure-care settings. These consequences involve youth mistrust of staff and the system, a fatalistic view that the legal system is not for them, and the feeling that they are on their own. From the perspective of vulnerability theory, these experiences mirror the child’s dependency on a secure-care institution and their personnel—institutions whose aim is to protect the child and provide resilience. If the children are not accepted and included in the societal system in which they are placed, on the same terms as other persons in society, they are removed from the universal and constant vulnerability to which all humans are subject. The ontology of the vulnerable subject does not accept different positions of some groups of individuals, independent of the institutional settings where they live. The theory of human vulnerability highlights the responsibility of social institutions such as secure-care institutions to function justly. This responsibility includes the obligation to balance the interests of all, including the children living in such institutions.

Hence, empirical results from our interviews of young persons fit well with Fineman’s vulnerability theory. Our conclusion from the empirical material—that children and young persons’ perceptions of care and treatment are strongly linked to their relationship to staff and how staff relate to and use restrictive measures—underlines these children and youths’ extensive dependency. The extent to which institution personnel, in their daily contacts with children, view children as bearers of rights and persons with full value appears to be closely connected to how youth perceive the behavior of personnel and the value of their stay and treatment. Thus, to achieve resilience, any analysis to determine what is non-harmful behavior in each situation must include a study of the children’s everyday life and not only their complaints and appeals of restrictive-measure decisions. This demands an analysis separate from an assessment of the position of some categories of children and youths to the institutional settings in which they live their day-to-day lives. In Sweden, this still remains to be done.

## Footnotes

1 This paper is part of an interdisciplinary project (law and social work), where the three researchers investigate compulsory care conducted by the Swedish National Board of Institutional Care (SiS). SiS’s activities are disputed in Sweden, not least owing to the forceful restrictive measures that SiS applies to the children assigned to the authority’s care. The overall purpose of the project is to analyze the effects of increased requirements to consider children's and young persons’ rights, interests and wishes in a compulsory-care context.

2 In this article, we use the terms *young persons* and *youth* to refer to persons between 18 and 20 years of age—the age limit for compulsory youth care according to the Swedish Care of Young Persons (Special Provisions) Act (SFS 1990:52). The act gives social services the authority to act to protect children and young persons under the age of 21.

3 In this article, we define *seclusion* by using the definition developed by young persons and practitioners in secure settings in the Netherlands. Seclusion is defined as: ‘an involuntary placement in a room or area the client is not able or allowed to leave’ [16].

4 Child welfare refers to the policy and practice of providing support and protection to children and young people, see Nancy Freymond and Gary Cameron, eds., *Towards Positive Systems of Child and Family Welfare. International Comparisons of Child Protection, Family Service, and Community Caring Systems* (Toronto: University of Toronto Press, 2006).

5 In addition to the compulsory social care as discussed in this article, children aged 15–17 and young people over 18 years, after having been sentenced for having committed serious criminal offences, may have their freedom of movement restricted in secure youth care under the Secure Youth Care Act (LSU). Alternatively, they may be assigned to compulsory care in locked psychiatric-care wards under the Act (1991:1128) on compulsory psychiatric care (LPT).

6 Care of Young Persons Act (1990:52), Sect. 12.

7 Care of Young Persons Act (1990:52), Sects. 15–20 and 43.

8 Swedish Government Official Reports 2015:71 part B, p. 871.

9 The Parliamentary Ombudsman decision no. 6774–2017, p. 3.

10 The written motivations for a total of 1,788 decisions (from 2019 and 2020) to use solitary confinement were reviewed.

11 Chapter 2, Sect. 6 of the Swedish Instrument of Government.

12 The Parliamentary Ombudsman decision no. 6774–2017, p. 10.

13 The Health and Social Care Inspectorate is not obliged to investigate all matters brought to its attention and makes an independent assessment regarding matters to investigate. The assessment is partly based on information received from individuals and partly on other information about various activities available to the Health and Social Care Inspectorate.

14 Official note from the Health and Social Care Inspectorate (IVO), case number AY (classified).

15 Complaint to the Health and Social Care Inspectorate (IVO), case number C (classified).

16 Decision by the Health and Social Care Inspectorate (IVO), case number AD (classified).

17 Children’s rights and evidence based care. Conflicting goals in compulsory care for young people? FORTE, dnr 2019/0004.

18 The interviews lasted between 13 och 72 min. All interviews, except five, were conducted in a meeting room at the unit where they youths lived. Due to Covid-19, we were not allowed to visit one of institutions and had to interview five (girls) using the digital Zoom web meeting application.

19 https://www.stat-inst.se/var-verksamhet/vard-av-unga/barns-och-ungas-rattigheter/.

20 This study (Gröön, A. 2021) is part of this research project.
